# Altered bioavailability of epoxyeicosatrienoic acids is associated with conduit artery endothelial dysfunction in type 2 diabetic patients

**DOI:** 10.1186/s12933-019-0843-z

**Published:** 2019-03-18

**Authors:** Thomas Duflot, Lucile Moreau-Grangé, Clothilde Roche, Michèle Iacob, Julien Wils, Isabelle Rémy-Jouet, Anne-Françoise Cailleux, Matthieu Leuillier, Sylvanie Renet, Dongyang Li, Christophe Morisseau, Fabien Lamoureux, Vincent Richard, Gaëtan Prévost, Robinson Joannidès, Jérémy Bellien

**Affiliations:** 1 Department of Pharmacology, Rouen University Hospital, 76000 Rouen Cedex, France; 2 Normandie Univ, UNIROUEN, INSERM U1096, FHU REMOD-VHF, 76000 Rouen, France; 3 Laboratory of Pharmacokinetics, Toxicology and Pharmacogenetics, Rouen University Hospital, 76000 Rouen, France; 4 Department of Endocrinology, Rouen University Hospital, 76000 Rouen, France; 5 Department of Entomology and Nematology, and Comprehensive Cancer Center, University of California, Davis, Davis, CA 95616 USA; 6 Normandie Univ, UNIROUEN, INSERM U1239, 76000 Rouen, France; 7 Centre d’Investigation Clinique (CIC)-INSERM 1404, Rouen University Hospital, 76000 Rouen, France

**Keywords:** Type 2 diabetes, Endothelial dysfunction, Soluble epoxide hydrolase, Epoxyeicosatrienoic acids

## Abstract

**Background:**

This pathophysiological study addressed the hypothesis that soluble epoxide hydrolase (sEH), which metabolizes the vasodilator and anti-inflammatory epoxyeicosatrienoic acids (EETs) to dihydroxyeicosatrienoic acids (DHETs), contributes to conduit artery endothelial dysfunction in type 2 diabetes.

**Methods and results:**

Radial artery endothelium-dependent flow-mediated dilatation in response to hand skin heating was reduced in essential hypertensive patients (n = 9) and type 2 diabetic subjects with (n = 19) or without hypertension (n = 10) compared to healthy subjects (n = 36), taking into consideration cardiovascular risk factors, flow stimulus and endothelium-independent dilatation to glyceryl trinitrate. Diabetic patients but not non-diabetic hypertensive subjects displayed elevated whole blood reactive oxygen species levels and loss of NO release during heating, assessed by measuring local plasma nitrite variation. Moreover, plasma levels of EET regioisomers increased during heating in healthy subjects, did not change in hypertensive patients and decreased in diabetic patients. Correlation analysis showed in the overall population that the less NO and EETs bioavailability increases during heating, the more flow-mediated dilatation is reduced. The expression and activity of sEH, measured in isolated peripheral blood mononuclear cells, was elevated in diabetic but not hypertensive patients, leading to increased EETs conversion to DHETs. Finally, hyperglycemic and hyperinsulinemic euglycemic clamps induced a decrease in flow-mediated dilatation in healthy subjects and this was associated with an altered EETs release during heating.

**Conclusions:**

These results demonstrate that an increased EETs degradation by sEH and altered NO bioavailability are associated with conduit artery endothelial dysfunction in type 2 diabetic patients independently from their hypertensive status. The hyperinsulinemic and hyperglycemic state in these patients may contribute to these alterations.

*Trial registration* NCT02311075. Registered December 8, 2014.

**Electronic supplementary material:**

The online version of this article (10.1186/s12933-019-0843-z) contains supplementary material, which is available to authorized users.

## Background

Despite standard treatments, the endothelial dysfunction of conduit arteries persists in type 2 diabetic patients and remains a main contributor of increased cardiovascular morbidity and mortality in this population, representing thus a critical therapeutic target [1, 2]. The mechanisms identified in animal models have been poorly assessed in humans and mainly depend on a chronic increased oxidative stress leading to nitric oxide (NO) degradation [1, 2]. Besides NO, we and others have shown in animal models of insulin resistance and diabetes that a decreased in epoxyeicosatrienoic acids (EETs) availability may be a main contributor to this endothelial dysfunction [3, 4]. There are four EET regioisomers (5,6-EET, 8,9-EET, 11,12-EET and 14,15-EET) synthesized from arachidonic acid by endothelial cytochrome P450 (CYPs), which display powerful anti-inflammatory and vasodilator properties, in animals and in human conduit arteries [5–7]. EETs are metabolized to the less active dihydroxyeicosatrienoic acids (DHETs) by soluble epoxide hydrolase (sEH), which is the target of a new class of pharmacological inhibitors [5, 6]. Interestingly, sEH inhibitors have been shown to improve endothelial function but also metabolic homeostasis in animal models of diabetes [3, 8], and correlation studies suggested that the more EETs bioavailability is elevated the less insulin sensitivity is altered in humans [9]. However, no study has investigated whether the alteration of EETs pathway is involved in the endothelial dysfunction of diabetic patients, which could strengthen the interest of using sEH inhibitors in this population.

The aim of the present study was to assess the mechanistic involvement of the EET/sEH pathway in the endothelial dysfunction of conduit arteries in type 2 diabetes in particular using biological approaches based on the quantification of endothelium-derived factors at baseline and during sustained blood flow stimulation induced by hand skin heating. Because essential hypertension is associated with an altered EETs bioavailability in conduit arteries [7, 10], we compared healthy subjects and patients with hypertension and/or type 2 diabetes, and performed complementary analyses in the overall study population to specifically address the impact of each of them. In addition, hyperglycemic and hyperinsulinemic clamps were performed to assess the proper impact of the elevation in glucose and/or insulin levels on endothelial function.

## Methods

### Population

This study was performed in a total of 74 subjects. Because the presence of hypertension may affect the evaluation of EETs pathway [7, 10], this cohort was divided in 4 groups as follow: healthy subjects, essential hypertensive subjects, type 2 diabetic subjects without hypertension and type 2 diabetic subjects with hypertension (Table 1). Subjects who smoked more than 5 cigarettes per day, with cardiac and/or cerebrovascular ischemic vascular disease, heart failure or impaired renal function (estimated glomerular filtration rate < 60 mL/min/1.73 m^2^) were excluded from the study. Patients with type 2 diabetes must not have a HbA1c > 9.4 mmol/L (7.5%), obtained with lifestyle management and standard hypoglycaemic agents except insulin.

**Table 1:** Clinical characteristics of the study population

Parameters	Healthy (n = 36)	Hypertension (n = 9)	Type 2 diabetes (n = 10)	Type 2 diabetes + hypertension (n = 19)
Age, years	54 ± 7	57 ± 9	58 ± 6	61 ± 6*
Male, n (%)	17 (47%)	5 (56%)	3 (33%)	13 (68%)
Body mass index (kg/m^2^)	25.2 ± 3.3	26.4 ± 4.2	29.4 ± 4.8*	30.9 ± 4.4*^†^
Smoking status current/past/never, n	3/7/26	0/0/9	1/2/7	0/11/8*^†^
SBP, mmHg	128 ± 9	140 ± 11*	134 ± 10	140 ± 15*
DBP, mmHg	80 ± 8	87 ± 5	83 ± 9	82 ± 10
MBP, mmHg	96 ± 1	104 ± 2*	100 ± 3	101 ± 2
Heart rate, bpm	67 ± 11	64 ± 13	71 ± 11	72 ± 10
LDL cholesterol, mg/dL	119 ± 46	125 ± 36	116 ± 25	87 ± 30*
HDL cholesterol, mg/dL	81 ± 41	58 ± 17	61 ± 25	53 ± 16*
Triglycerides, mg/dL	94 ± 40	116 ± 53	135 ± 68	117 ± 43
Fasting glucose, mg/dL	93 ± 9	97 ± 12	142 ± 22*^†^	130 ± 28*^†^
Insulinemia,	58 ± 31	91 ± 54	98 ± 55	121 ± 73*
Hb1Ac, mmol/L	–	–	8.8 ± 0.7	8.3 ± 0.7
TyG index	4.50 ± 0.22	4.62 ± 0.24	4.85 ± 0.27*	4.79 ± 0.19*
Creatinemia, µmol/L	73 ± 12	78 ± 19	63 ± 14	80 ± 12
Urinary ACR, mg/mmol	0.72 ± 0.46	1.23 ± 0.67	1.33 ± 1.19	1.31 ± 0.96
Blood viscosity, cP	4.3 ± 0.5	4.4 ± 0.7	4.4 ± 0.6	3.9 ± 0.5
Statins, n (%)	1 (3%)	3 (33%)*	2 (20%)	14 (74%)*^‡^
Antihypertensive agents, n (%)				
ACEi/ARB	–	7 (78%)	–	14 (74%)
CCB	–	2 (20%)	–	12 (63%)
Beta-blockers	–	1 (11%)	–	2 (11%)
Diuretics	–	0 (0%)	–	8 (42%)^†^
Hypoglycemic agents, n (%)				
Metformin	–	–	10 (100%)	15 (79%)
Sulfamides/glinides	–	–	2 (20%)	8 (42%)
DPP-4 inhibitors/GLP-1 agonists	–	–	4 (40%)	9 (47%)

Data are mean ± SD or n (%)

*ACEi* angiotensin-converting enzyme inhibitors, *ACR* albumin-to-creatinine ratio, *ARB* angiotensin type 1 receptor blockers, *CCB* calcium channel blockers, *DBP* diastolic blood pressure, *DPP-4* dipeptidyl peptidase-4, *GLP-1* glucagon-like peptide-1, *MBP* mean blood pressure, *SBP* systolic blood pressure, *TyG* triglyceride-glucose index

* *P *< 0.05 vs. healthy

^†^*P *< 0.05 vs. hypertension

^‡^*P *< 0.05 vs. type 2 diabetes

### Assessment of endothelial function

Radial artery endothelium-dependent flow-mediated dilatation was assessed using the previously validated hand skin heating technique, from 34 to 44 °C, allowing to induce a progressive and sustained increase in blood flow [7, 10, 11]. Radial artery endothelium-independent dilatation, corresponding to the ability of smooth muscle cells to relax in response to exogenous NO, was assessed then using 0.3 mg sublingual glyceryl trinitrate (GTN). See the Additional file 1 for further details.

### Biological parameters

A 4-F catheter was inserted into the forearm cephalic vein, when accessible, allowing blood sampling in the venous return at 34 and 44 °C for the quantification of the endothelial factors that have shown to be involved in flow-mediated dilatation [7]. Quantification of plasma 8,9-EET, 11,12-EET, 14,15-EET and corresponding DHET regioisomers was performed by a recently validated method based on lipid extraction and saponification followed by liquid chromatography coupled to tandem mass spectrometry analysis (LC–MS/MS) [10]. Total EETs + DHETs levels and ratio of the preferential substrate of sEH 14,15-EET to its metabolite 14,15-DHET was used as an index of EETs production and sEH activity respectively [10]. In addition, the levels of epoxy fatty acids synthesized by CYPs from linoleic acid and from the omega-3 docosahexaenoic acid (DHA): 9,10- and 12,13-epoxyoctadecenoic (9,10-EpOME and 12,13-EpOME), 19,20-epoxydocosapentaenoic acid (19,20-EpDPA) and their respective diols produced by sEH, 9,10-, 12,13-dihydroxyoctadecenoic acid (9,10-DiHOME and 12,13-DiHOME) and 19,20-dihydroxydocosapentaenoic acid (19,20-DiHDPA), were quantified by LC–MS/MS. Quantification of the plasma level of the NO metabolite nitrite was performed by a tri-iodide/ozone-based chemiluminescence assay [7]. Additional blood sampling was performed at 34 °C to quantify the whole blood level of reactive oxygen species (ROS) by electron paramagnetic resonance spectroscopy (Miniscope MS-200, Magnettech) [7]. In addition, peripheral blood mononuclear cells (PBMCs) were isolated by density gradient centrifugation in order to determine sEH protein expression and activity using an ultrasensitive nanobody polymeric horseradish peroxidase-based immunoassay and a [^3^H]-trans-1,3-diphenylpropene oxide-based radioactivity assay respectively [12, 13]. Results were normalized to total protein concentration in PBMCs. In addition, mRNA expression levels of sEH and of the main CYPs involved in the vascular production of EETs CYP2C9 and CYP2C19 were determined in PBMCs by quantitative RT-PCR (primers are shown in Additional file 1: Table S1). Absolute abundance of mRNA relative to beta2-microglobulin housekeeping gene expression was calculated based on a cDNA standard curve. See Additional file 1 for further details.

### Hyperglycemic and hyperinsulinemic clamps

Hyperglycemic and hyperinsulinemic euglycemic clamps were performed in 8 subjects selected from the healthy group (mean [SD] age: 46 ± 5 years, 6 male subjects) and explored on two separate occasions after a 12 h overnight fasting. Briefly, a venous catheter was inserted in a large vein at the antecubital fossa of the dominant arm, allowing a variable infusion rate of a 20% glucose solution alone or combined with continuous regular insulin administration (Actrapid^®^ 100 UI/mL, Novo Nordisk: 250, 200, 150, 100 mU/m^2^ of body surface area [BSA] per min for 2 min each, and then 80 mU/m^2^ of BSA per min) to achieve and maintain either a target steady-state capillary glucose concentration of 11 mmol/L (200 mg/dL) for the hyperglycemic clamp or 5.5 mmol/L (100 mg/dL) for the hyperinsulinemic clamp, without change in glucose infusion rate for at least 30 min before assessing radial artery endothelium-dependent and -independent dilatation. A control procedure was also performed with saline infusion. Blood sampling were performed before beginning the clamp, at glycemic steady-state before hand skin heating and at the end of hand skin heating to determine the evolution of nitrite, EETs and DHETs bioavailability.

### Statistical analysis

Analyses were performed using NCSS software (version 07.1.14) and R software (version 3.5.1). The Shapiro–Wilk test was used to assess normality of data.

Differences between healthy, hypertensive, type 2 diabetic and hypertensive type 2 diabetic groups were assessed by Fisher’s exact test for categorical variables and by one-way ANOVA or nonparametric Kruskall–Wallis test for continuous variables, followed in case of significance, by Tukey’s test or Dunn’s test for multiple comparisons.

Significant predictors for radial artery flow-mediated dilatation (percent change in radial artery diameter from 34 to 44 °C) were assessed using both backward and forward stepwise regression based upon Akaike Information Criterion (AIC) using a null model without predictors and a full model including diabetes, hypertension, sex, age, smoking status, mean arterial blood pressure, BMI, LDL cholesterol level, triglyceride-glucose (TyG) index (log [fasting triglycerides (mg/dL) × fasting glucose (mg/dL)/2]), creatininemia, baseline radial diameter, variation in shear stress and GTN-induced dilatation as predictors. Similar analyses were performed for shear stress variation during heating and GTN-induced dilatation.

Pearson correlation analyses were performed to determine linear relationships between biological and functional variables of interest.

Differences between the impact of saline infusion, hyperglycemic clamp and hyperinsulinemic clamp on the variations in radial artery parameters at baseline and during heating and on the variations in the increase in diameter following GTN administration were assessed by ANOVA using a generalized linear model with group and subject as factors followed, in case of significance, by a Tukey’s test for posthoc pairwise comparisons. The impact of the hyperglycemic and the hyperinsulinemic euglycemic clamps on biological parameters at baseline and during heating were assessed by paired t-test or Wilcoxon matched pairs test. A value of *P *≤ 0.05 was considered statistically significant.

## Results

### Comparative approach

#### Baseline characteristics

Baseline characteristics of the subjects included are summarized in Table 1. There was no difference between groups for sex ratio, diastolic blood pressure, heart rate, triglycerides levels, creatinemia, blood viscosity and urinary albumin-to-creatinine ratio. Age was higher in the hypertensive type 2 diabetic subjects compared to the healthy subjects. As expected, BMI and glycemia were higher in the type 2 diabetic groups compared with the non-diabetic groups. Insulinemia was higher in the hypertensive diabetic group compared with the healthy group. Moreover, TyG, a marker of insulin resistance was higher in the 2 diabetic groups compared with the healthy subjects. Furthermore, subjects with hypertension, with and without association to type 2 diabetes, had an increased systolic blood pressure but only essential hypertensive patients had an increased mean blood pressure. Finally, hypertensive type 2 diabetic subjects were more frequently treated with statins and had a lesser LDL and HDL cholesterol levels compared to the healthy subjects.

#### Radial artery endothelium-dependent and -independent dilatation

**Fig. 1:**
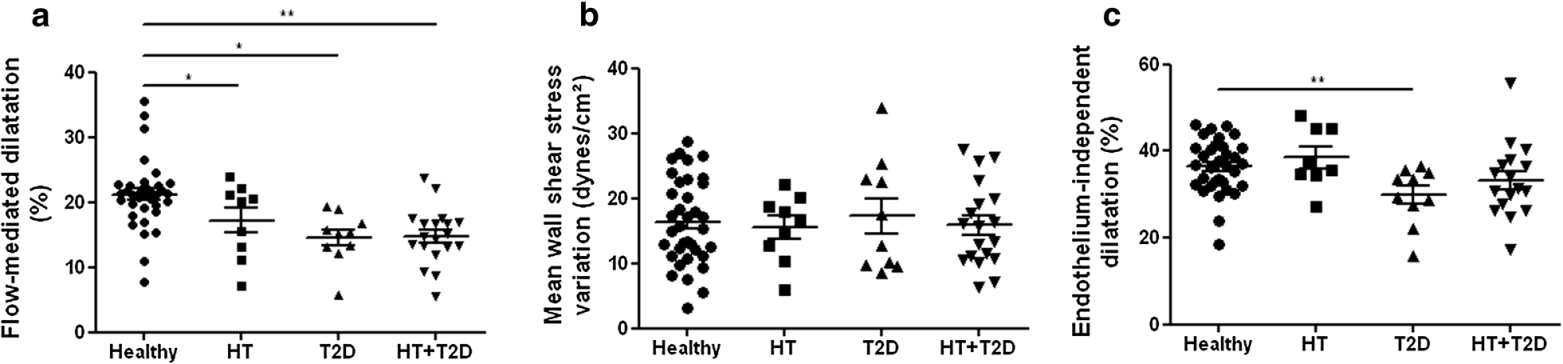
Presence of conduit artery endothelial dysfunction in type 2 diabetes and essential hypertension. Variations in radial artery diameter (**a**) and mean wall shear stress (**b**) in response to hand skin heating, and variation in radial artery diameter in response to glyceryl trinitrate (**c**) in healthy, hypertensive (HT), type 2 diabetic (T2D) and hypertensive type 2 diabetic (HT + T2D) subjects. Mean values ± SEM are shown. *P < 0.05, **P < 0.01

There was no significant difference between groups for baseline radial artery diameter and mean wall shear stress assessed at 34 °C before heating (Additional file 1: Table S2). During hand skin heating, compared to healthy subjects, the radial artery flow-mediated dilatation was reduced in essential hypertensive patients and in type 2 diabetic subjects independently of their hypertensive status (Fig. 1a), taking into consideration their associated cardiovascular risk factors, the flow stimulus and smooth muscle reactivity (Additional file 1: Table S3). In fact, the variation in mean wall shear stress was similar between groups but the endothelium-independent dilatation to GTN was reduced in diabetic patients without hypertension (Fig. 1b, c; Additional file 1: Table S3). Put together, these results demonstrate the presence of a conduit artery endothelial dysfunction in essential hypertensive patients and in type 2 diabetic patients.

#### Biochemical evaluation of NO and EETs pathways

**Fig. 2:**
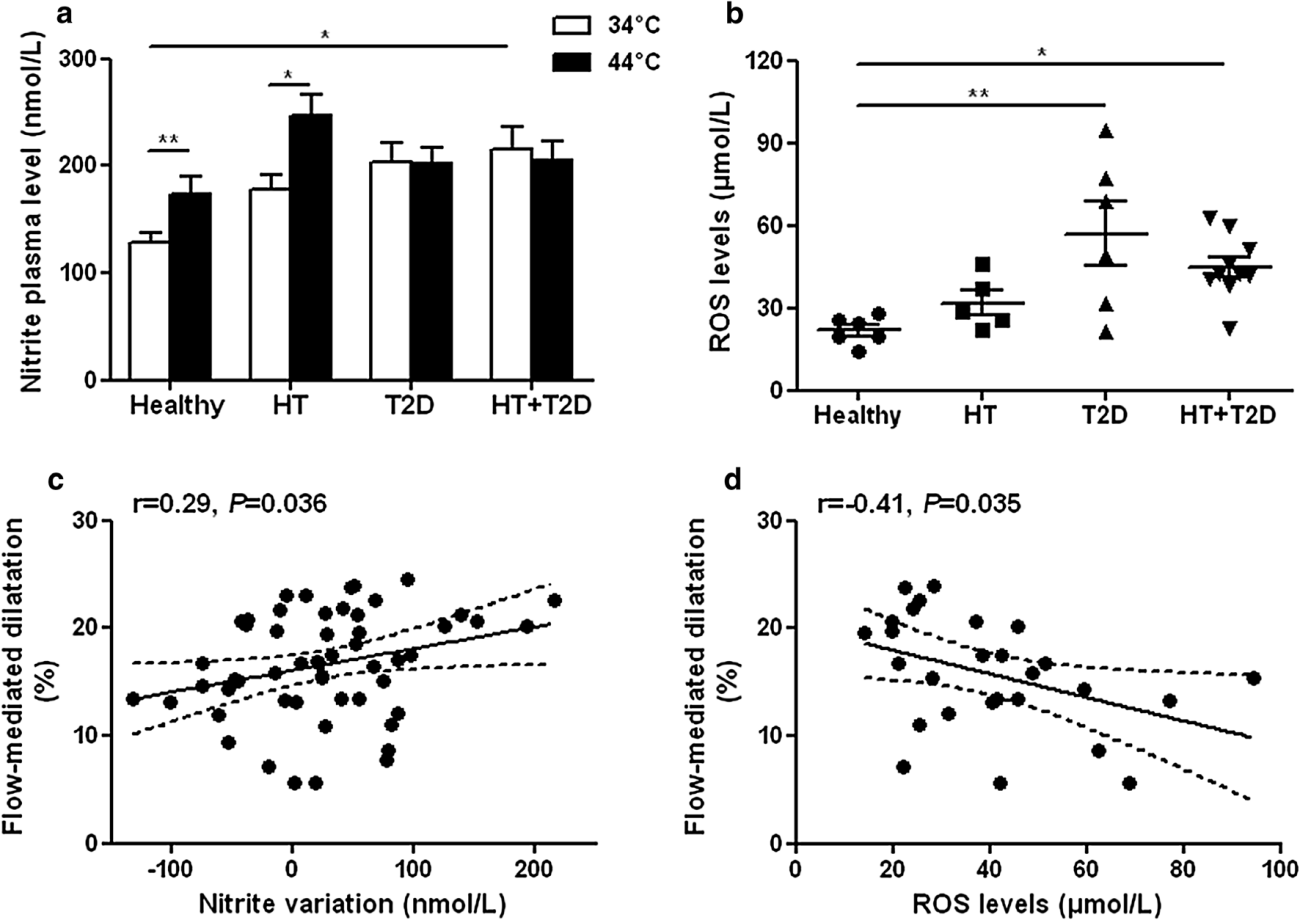
Altered NO bioavailability in type 2 diabetes. Plasma levels of the NO metabolite nitrite (**a**) before (34 °C) and at the end of hand skin heating (44 °C) in healthy (n = 23), hypertensive (HT; n = 6), type 2 diabetic (T2D; n = 8) and hypertensive type 2 diabetic (HT + T2D; n = 18) subjects. Whole blood levels of reactive oxygen species (ROS) (**b**) in healthy, hypertensive (HT), type 2 diabetic (T2D) and hypertensive type 2 diabetic (HT + T2D) subjects. Mean values ± SEM are shown. *P < 0.05, **P < 0.01. Linear relationships between the magnitude of radial artery flow-mediated dilatation with the variations in plasma nitrite levels during heating (**c**) and with ROS levels (**d**). The dashed lines represent the 95% confidence interval for the regression

The plasma nitrite levels were higher at baseline (34 °C) in type 2 diabetic patients and this was statistically significant in hypertensive diabetic patients compared to healthy subjects. Plasma nitrite increased during heating in healthy and hypertensive subjects but not in the diabetic groups (Fig. 2a), showing an altered NO release during the endothelial stimulation. This was associated with increased ROS whole blood levels in the 2 diabetic groups compared with the healthy group (Fig. 2b). Correlation analyses show in the overall study population that the magnitude of radial artery flow-mediated dilatation was positively associated with the variations in nitrite levels during heating (Fig. 2c) and inversely correlated with ROS levels (Fig. 2d).

**Fig. 3:**
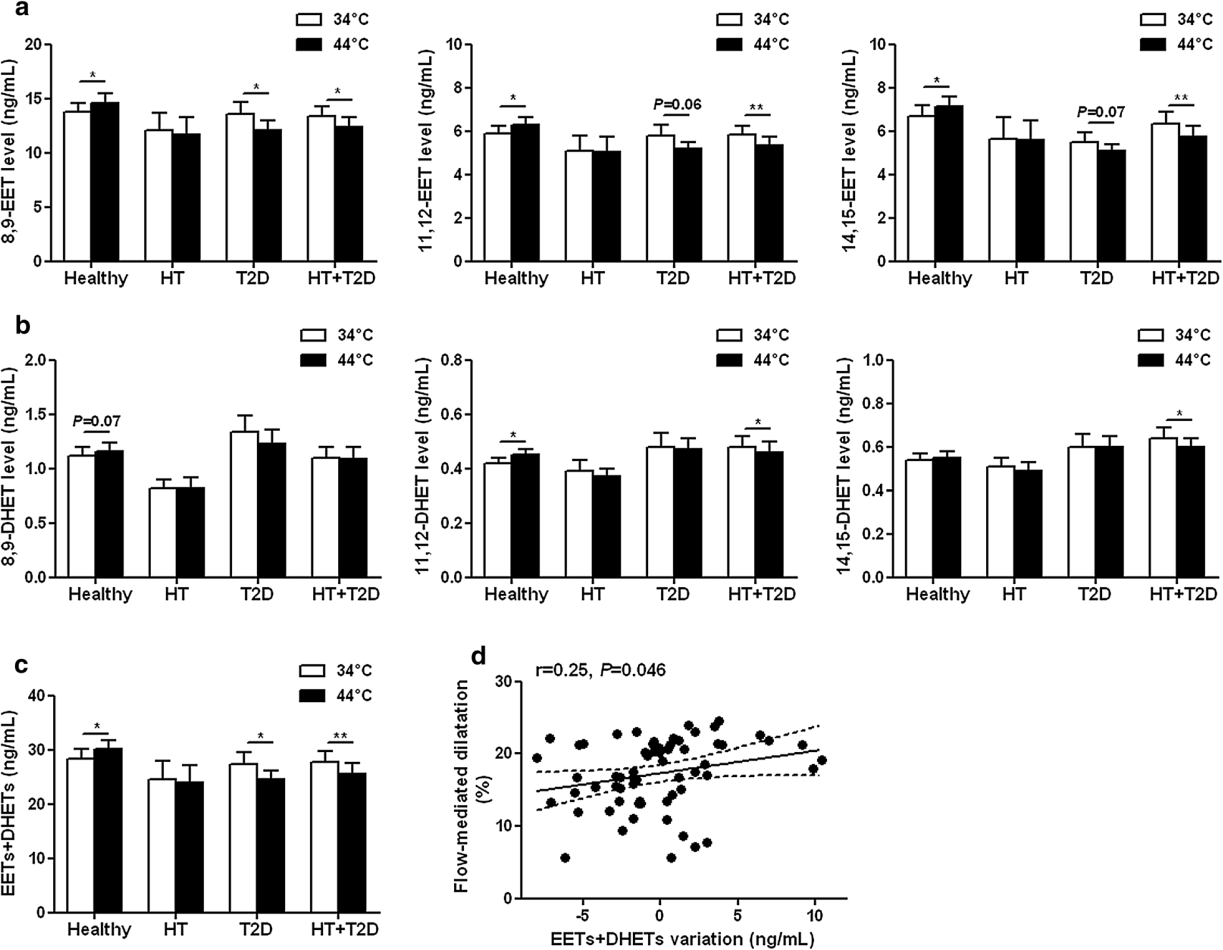
Type 2 diabetes profoundly impaired EETs bioavailability during endothelial stimulation. Plasma levels of epoxyeicosatrienoic acids (EETs) (**a**), dihydroxyeicosatrienoic acids (DHETs) (**b**) regioisomers and total EETs + DHETs levels (**c**) before (34 °C) and at the end of hand skin heating (44 °C), in healthy (n = 28), hypertensive (HT; n = 8), type 2 diabetic (T2D; n = 9) and hypertensive type 2 diabetic (HT + T2D; n = 19) subjects. Mean values ± SEM are shown. *P < 0.05, **P < 0.01. Linear relationships between the magnitude of radial artery flow-mediated dilatation with the variations in plasma EETs + DHETs during heating (**d**). The dashed lines represent 95% confidence interval for the regression

**Fig. 4:**
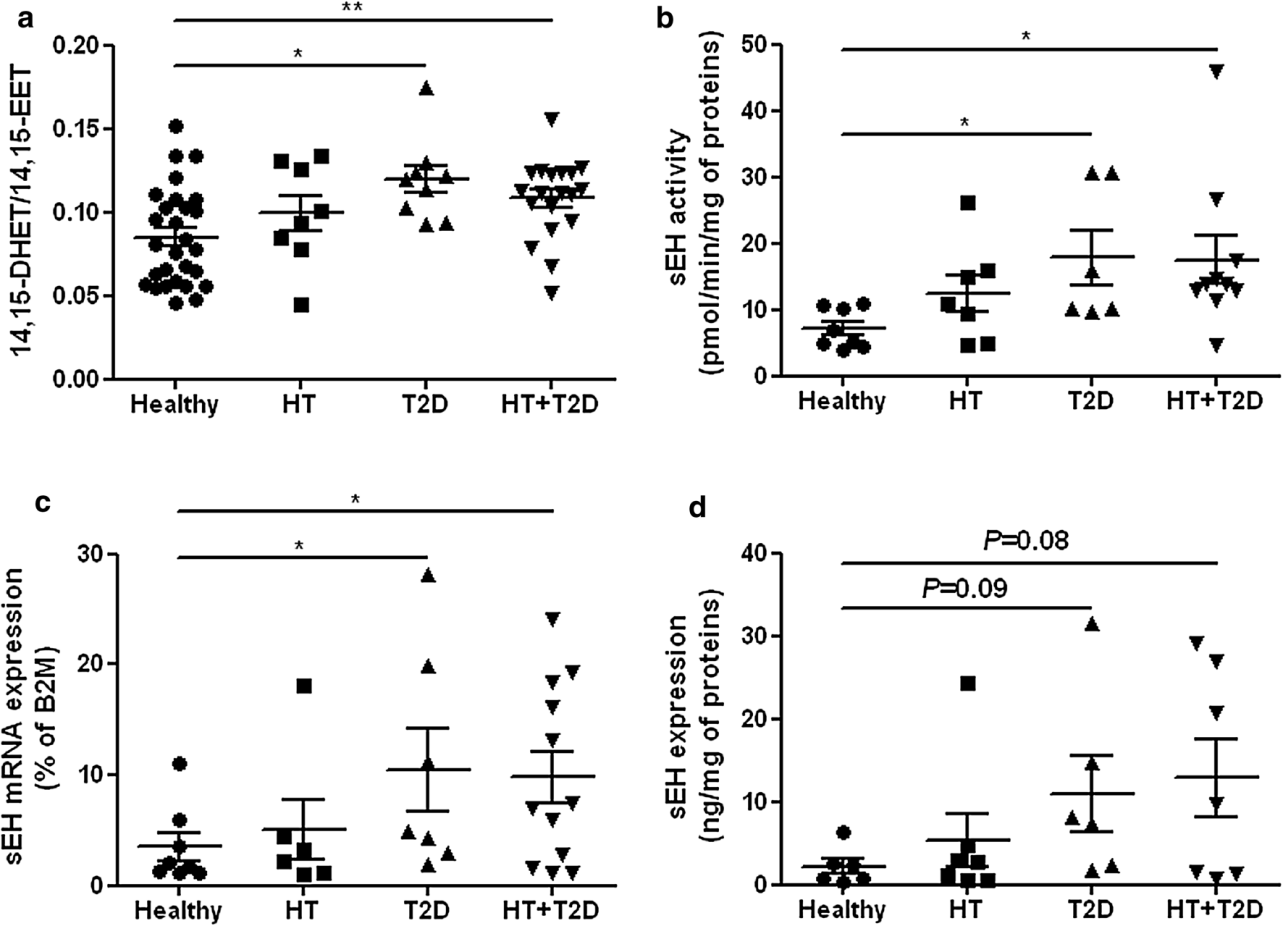
Increased EETs degradation by sEH in type 2 diabetes. Ratio of plasma 14,15-dihydroxyeicosatrienoic acid-to-14,15-epoxyeicosatrienoic acid (14,15-DHET/14,15-EET) (**a**), sEH activity (**b**), mRNA (**c**) and protein expression (**d**) in peripheral blood mononuclear cells in healthy, hypertensive (HT), type 2 diabetic (T2D) and hypertensive type 2 diabetic (HT + T2D) subjects. β2M: beta2-microglobulin. Mean values ± SEM are shown. *P < 0.05, **P < 0.01

Regarding the EETs pathway, no difference was found between groups in the baseline plasma levels of EET (Fig. 3a) or DHET (Fig. 3b) regioisomers nor in total EETs + DHETs levels (Fig. 3c). The levels of most of the EET and DHET regioisomers and subsequently total EETs + DHETs levels increased during heating in the healthy subjects but did not change in the hypertensive subjects and even decreased in the type 2 diabetic groups (Fig. 3a–c), illustrating an altered EET production during the endothelial stimulation. However, we could not determine whether differences between groups exist regarding the expression of the CYPs involved in EETs production because they cannot be detected in human PBMCs. Correlation analyses show in the overall study population that the magnitude of radial artery flow-mediated dilatation was positively associated with the variations in EETs + DHETs (Fig. 3d). Moreover, there was an increased conversion of EETs to DHETs in the type 2 diabetic groups, as shown by their higher 14,15-DHET-to-14,15-EET ratio at the end of heating compared to healthy subjects (Fig. 4a). The increased sEH activity in type 2 diabetic patients was directly demonstrated in isolated PBMCs (Fig. 4b) and appeared mainly related to the upregulation of sEH mRNA (Fig. 4c) and protein (Fig. 4d) expression level. Accordingly, there is a significant positive correlation between sEH protein expression and activity (Additional file 1: Figure S1a). In addition, sEH activity is positively associated with the plasma level of the main diol formed by sEH 14,15-DHET (Additional file 1: Figure S1b) and negatively associated with the variation in EETs during heating (Additional file 1: Figure S1c) and flow-mediated dilatation (Additional file 1: Figure S1d). Furthermore, plasma levels of 19,20-EpDPA, 9,10-EpOME, 12,13-EpOME and corresponding diols did not change during heating and the diol-to-epoxide ratios remained similar between groups (Additional file 1: Figure S2a–c).

### Metabolic approach

The hyperglycemic clamp increased plasma glucose and insulin concentrations while the hyperinsulinemic clamp increased insulin levels to a higher level, without significantly affecting glucose level (Additional file 1: Figure S3a, b).

**Fig. 5:**
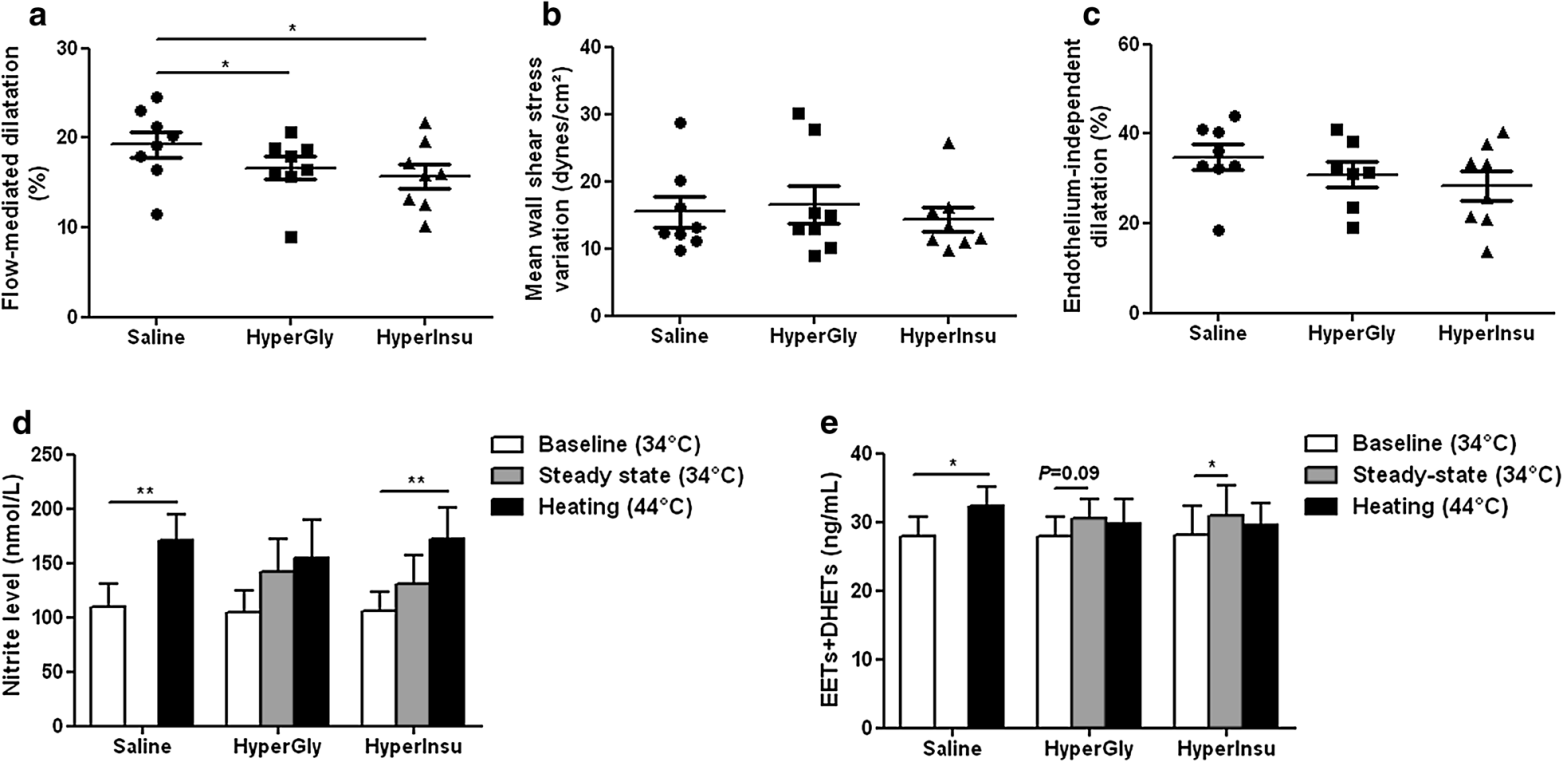
Acute hyperglycemia and hyperinsulinemia altered conduit artery endothelial function in healthy subjects. Variations in radial artery diameter (**a**) and mean wall shear stress (**b**) in response to hand skin heating and variation in radial artery diameter in response to glyceryl trinitrate (**c**) during saline infusion an during the hyperglycemic (HyperGly) and hyperinsulinemic euglycemic (HyperInsu) clamps in 8 healthy subjects. Plasma levels of nitrite (**d**) and total epoxyeicosatrienoic acids + dihydroxyeicosatrienoic acids (EETs + DHETs) (**e**) at baseline at 34 °C, at steady-state and at the end of hand skin heating at 44 °C during saline infusion and during the hyperglycemic and hyperinsulinemic euglycemic clamps in 8 healthy subjects. Mean values ± SEM are shown. *P < 0.05, **P < 0.01

Compared to saline infusion, the basal radial artery diameter was increased by both clamps at steady-state before hand skin heating but statistical significance was only reached with the hyperinsulinemic clamp, without change in mean wall shear stress and in systemic hemodynamics (Additional file 1: Table S4). Radial artery flow-mediated dilatation in response to heating (Fig. 5a) was reduced by both clamps, without change in mean wall shear stress variation (Fig. 5b) nor in endothelium-independent dilatation to GTN (Fig. 5c).

The plasma nitrite levels were not significantly modified by both clamps at steady-state before hand skin heating (Fig. 5d). Nitrite levels increased during hand skin heating in presence of saline, was not modified during the hyperglycemic clamp but increased compared to baseline conditions during the hyperinsulinemic clamp (Fig. 5d). In addition, the hyperinsulinemic clamp increased the plasma levels of EET regioisomers (Additional file 1: Figure S4a) without significant change in the levels of DHET regioisomers (Additional file 1: Figure S4b), resulting in an increase in total EETs + DHETs (Fig. 5e). In addition, the 14,15-DHET-to-14,15-EET ratio was decreased during the hyperinsulinemic clamp (Additional file 1: Figure S5). A trend for an increase in the levels of EET regioisomers was also observed during the hyperglycemic clamp but there was no change in the 14,15-DHET-to-14,15-EET ratio (Fig. 5e; Additional file 1: Figures S4 and S5). Heating induced an increase in the plasma levels of EETs and DHETs in presence of saline but not during both clamps and even, EETs and DHETs became similar to baseline conditions (Fig. 5e; Additional file 1: Figure S4).

## Discussion

The major finding of the present work is that the alteration of conduit artery endothelial function in type 2 diabetic patients was partly due to an alteration of the EETs pathway, in parallel to the expected contribution of the decreased NO availability.

### Endothelial dysfunction is present in essential hypertensive and type 2 diabetic patients

In this study, we carefully selected type 2 diabetic patients treated according to recent recommendations for optimization of glycemic control together with management of associated risk factors, mainly LDL cholesterol and blood pressure. To evaluate the proper impact of type 2 diabetes independently from the rise in blood pressure, we compared the functional and biological data of healthy, essential hypertensive, type 2 diabetic and hypertensive type 2 diabetic subjects.

In this context, we demonstrated that type 2 diabetic patients either in absence or in presence of hypertension display a decreased sustained radial artery flow-mediated in response to heating, taking into consideration cardiovascular risk factors, the flow-dependent stimulus and endothelium-independent dilatation to GTN. These results demonstrate that well-controlled type 2 diabetic patients still exhibit an endothelial dysfunction of peripheral conduit arteries, as previously shown using more transient increase in blood flow induced by post-ischemic hyperemia [1, 2, 14–16]. In addition, a decreased endothelium-independent dilatation in response to exogenous NO was also observed in type 2 diabetic patients but only in absence of hypertension, suggesting that antihypertensive agents may contribute to prevent smooth muscle cell dysfunction in type 2 diabetes. To note, the decrease in the flow-mediated dilatation of patients with treated hypertension alone appeared less marked but was statistically significant when compared to healthy subjects, taking into consideration cardiovascular risk factors. In fact, decreasing blood pressure per se allows to normalize endothelial function in less than half of the patients and some antihypertensive medications, in particular blockers of the renin-angiotensin system, have direct protective effects on vascular function [7, 17, 18].

### Role of altered NO bioavailability in endothelial dysfunction

Regarding the NO pathway, the basal nitrite level was elevated but did not further increase during the sustained endothelial stimulation in the two groups of diabetic patients. This may be related to the increase in oxidative stress in diabetic patients illustrated by the elevation in the whole blood levels of ROS. Indeed, previous work suggested that hyperglycemia may lead to an increased expression of endothelial NO-synthase to compensate for the enhanced inactivation of NO by superoxide anions and promoting increased peroxynitrite formation [19, 20]. A parallel increase in the expression of inducible NO-synthase related to the pro-inflammatory phenotype associated with type 2 diabetes may exist [20]. Part of the NO synthesized in excess is not degraded but converted to nitrite explaining the increased basal level [19]. However, the absence of increase in nitrite level during heating demonstrate an impairment in stimulated NO bioavailability at the level of conduit arteries in type 2 diabetes. As supported by correlations analyses, the NO/ROS imbalance may contribute to the observed reduction in endothelium-dependent dilatation of conduit arteries in type 2 diabetic patients. Accordingly, a decrease in NO bioavailability was previously reported in forearm resistance arteries using functional approaches based on the local infusion of the NO-synthase inhibitor L-NMMA during endothelial stimulation [21, 22]. In contrast, patients with treated essential hypertension still had an increase in NO level during heating and no change in ROS levels. Thus, while non-treated hypertensive patients have a reduced NO bioavailability [7], this maintained response in subjects mainly treated with blockers of the renin-angiotensin system may contribute to partially preserve the endothelial vasomotor function.

### Role of altered EETs pathway in endothelial dysfunction

Furthermore, our results demonstrate for the first time in humans that the endothelial dysfunction of conduit arteries in type 2 diabetes is associated with an alteration in the EETs pathway. In fact, according with previous clinical studies [7, 23, 24], we cannot detect a significant difference between groups for the baseline plasma levels of EET and DHET regioisomers but this may be due to the limited number of patients in the hypertensive and diabetic groups. However, we can demonstrate that, in contrast to what is observed in the healthy subjects, EETs did not increase during the endothelial stimulation in hypertensive patients, as previously shown in both treated and non-treated subjects [7, 10], and they even decreased in the 2 groups of diabetic patients. Although the magnitude of the increase in EETs in healthy subjects appears modest, we observed using functional inhibition of CYPs that a similar magnitude of the variation in EETs during heating can contribute up to one-third of the endothelial-dependent dilatation in response to the sustained flow increase [7]. Thus, because plasma levels of DHETs and thus EETs + DHETs also decreased during heating in type 2 diabetic patients, we can speculate that the production of EETs cannot increase in response to shear stress contributing, as suggested from correlation analyses, to the endothelial vasomotor dysfunction. In fact, increasing blood flow while keeping the same EET production than during baseline flow conditions has a diluting effect; this is probably the main explanation for the decrease in local concentrations of EETs and DHETs in diabetic patients. This alteration in EETs production may be related to a reduction in CYPs expression levels, as previously observed at the renal level in obese and in salt-sensitive Dahl rats and notably contributing to the development of high blood pressure [4, 25, 26]. Unfortunately, in accordance with previous results [27], we were not able to detect in PBMCs the expression of the main CYPs involved in the vascular production of EETs to confirm this hypothesis. In addition to the putative decrease in EETs production, our results show the presence of an increased conversion of EETs to DHETs by sEH. Indeed, we noticed an increased 14,15-DHET-to-14,15-EET ratio in plasma and directly demonstrated an increased sEH expression and activity in PBMCs of type 2 diabetic patients compared to healthy subjects. Although sEH measurements can only be performed non invasively in vivo in PBMCs, they probably reflect the evolution of sEH at the vascular level, which probably plays an important role in metabolizing the EETs released during the endothelial stimulation, preventing their relaxing effect on vascular smooth cells. This hypothesis is supported by the inverse correlations between sEH activity with EETs variation during heating and the magnitude of flow-mediated dilatation. This increased sEH expression/activity is consistent with most of the results obtained in animal models of obesity-induced insulin resistance [27–30]. Of note, the percentage of patients that were on blockers of the renin-angiotensin system was similar in the hypertensive and hypertensive type 2 diabetic groups and thus, these agents may have contributed to prevent the upregulation of sEH expression induced by angiotensin II in essential hypertensive subjects but not in type 2 diabetic patients [31].

In contrast, no change in the plasma levels of other epoxy fatty acids and diols derived from linoleic acid or omega-3 were observed, suggesting that their altered metabolism does not contribute to the endothelial dysfunction in our population of well-controlled type 2 diabetic patients. These results extent recent works showing that that the levels of omega-3 and linoleic acid appear no altered when glycemic control is achieved and that omega-3 supplementation is ineffective to improve the metabolic and inflammatory status of patients with well-controlled type 2 diabetes [32, 33]. Finally, as previously suggested [23, 34], we cannot exclude that an increase in the levels of the vasoconstrictor 20-hydroxyeicosatetraenoic (20-HETE) synthesized from arachidonic acid by CYPs hydroxylases contributes to the endothelial dysfunction of essential hypertensive and type 2 diabetic patients.

### Role of high glucose and high insulinemia in the development of endothelial dysfunction

Going further, we assessed the role of hyperglycemia and hyperinsulinemia in the development of endothelial dysfunction using clamp studies in healthy subjects. Both clamps slightly induced an increase in radial artery diameter, without significant change in mean wall shear stress, but this increase appears stronger with the euglycemic hyperinsulinemic clamp reaching statistical significance compared to basal conditions. This arterial vasodilating effect was probably related to the potentiation of endothelial factor release by insulin, although the increase in nitrite levels was not significant. Previous results showed that acute hyperglycemia or hyperinsulinemia dilates peripheral resistance arteries by a NO-dependent mechanism involving endothelial NO-synthase phosphorylation [35, 36], and our results suggest that this mechanism may be less efficient during more sustained hyperinsulinemia. In contrast, we observed that the levels of EET and DHET regioisomers were increased at steady-state during the euglycemic hyperinsulinemic clamps. To our knowledge, this is the first study showing directly in humans that insulin promotes the production of EETs and that this mechanism may contribute to the vasodilator effect and potentiate insulin sensitivity [9]. Moreover, we observed a marked decrease in the 14,15-DHET-to-14,15-EET ratio, suggesting that insulin may also decrease EETs degradation. Whether the transitional tyrosine phosphorylation of sEH, suggested to modulate its activity [37, 38], is involved in this effect of insulin remains to be determined.

Despite this basal vasodilator effects and as generally observed using post-ischemic hyperemia [39–44], hyperglycemia and hyperinsulinemia reduced the endothelium-dependent flow-mediated dilatation of peripheral conduit arteries but the mechanisms involved appear somewhat different. As suggested from the absence of increase in nitrite levels during heating, hyperglycemia may reduce local NO bioavailability during endothelial stimulation. This may be related to the increase in oxidative stress, as suggested from the reversion of endothelial dysfunction induced by hyperglycemia obtained with antioxidants [41, 42]. In addition, we did not observe an increase in EETs during the hyperglycemic clamp suggesting altered EETs production. For the euglycemic hyperinsulinemic clamp, the impact on stimulated NO availability appears less marked because nitrite levels finally increased in response to heating but only when compared to baseline conditions before the beginning of the clamp. However, EETs levels did not increase and even slightly decreased in response to heating. Thus, the difference between the basal conditions before the beginning of the clamp was no more statistically significant, demonstrating that a marked alteration in stimulated EETs production cannot compensate for the diluting effect of the increase in blood flow. The mechanism of the discrepancy between the impact of hyperinsulinemia at baseline and during flow stimulation on EETs production remain to be elucidated but insulin may promote the release of EETs stored in phospholipids pools while altering the de novo production of EETs by CYPs, needed for flow-mediated dilatation [7, 45].

## Conclusions

Altogether, our results demonstrate that a decreased EETs production and increased EETs degradation by sEH together with altered NO bioavailability are both associated with conduit artery endothelial dysfunction in type 2 diabetic patients on top of standard treatments. In contrast, treated essential hypertensive patients appeared to only display a decreased EETs production but maintained NO levels (Additional file 1: Figure S6). Although additional experiments are needed to clarify the mechanisms involved, the hyperglycemic and hyperinsulinemic state in type 2 diabetic patients may play a critical role in these alterations. Of importance, this clinical work extends previous findings in animal models of insulin-resistance, showing that sEH inhibition may help to prevent endothelial dysfunction [3, 8]. In fact, targeting sEH to increase protective epoxy fatty acids, such as EETs, but also to decrease the formation of pro-inflammatory diols derived from linoleic acid or omega-3 fatty acids in more advanced stage of the diabetic disease, represents a new pharmacological approach to treat the cardiovascular complications, the metabolic abnormalities and ocular complications of type 2 diabetes [3, 8, 30, 46, 47]. Interventional studies with newly developed sEH inhibitors are now needed to finally demonstrate that this pharmacological class displays such promising protective effects in type 2 diabetes.

## Additional file

**Additional file 1.** Supplementary Methods and Results.

## Abbreviations

CYP: cytochrome P450
DHET: dihydroxyeicosatrienoic acid
EET: epoxyeicosatrienoic acid
GTN: glyceryl trinitrate
LC–MS/MS: liquid chromatography coupled to tandem mass spectrometry analysis
PBMC: peripheral blood mononuclear cell
ROS: reactive oxygen species
sEH: soluble epoxide hydrolase

## References

[1] van Sloten TT, Henry RM, Dekker JM, Nijpels G, Unger T, Schram MT, Stehouwer CD (2014). Endothelial dysfunction plays a key role in increasing cardiovascular risk in type 2 diabetes: the Hoorn study. Hypertension.

[2] Sena CM, Pereira AM, Seiça R (2013). Endothelial dysfunction—a major mediator of diabetic vascular disease. Biochim Biophys Acta.

[3] Roche C, Besnier M, Cassel R, Harouki N, Coquerel D, Guerrot D, Nicol L, Loizon E, Morisseau C, Remy-Jouet I, Mulder P, Ouvrard-Pascaud A, Madec AM, Richard V, Bellien J (2015). Soluble epoxide hydrolase inhibition improves coronary endothelial function and prevents the development of cardiac alterations in obese insulin-resistant mice. Am J Physiol Heart Circ Physiol.

[4] Zhao X, Dey A, Romanko OP, Stepp DW, Wang MH, Zhou Y, Jin L, Pollock JS, Webb RC, Imig JD (2005). Decreased epoxygenase and increased epoxide hydrolase expression in the mesenteric artery of obese Zucker rats. Am J Physiol Regul Integr Comp Physiol.

[5] Bellien J, Joannides R, Richard V (2011). Modulation of cytochrome-derived epoxyeicosatrienoic acids pathway: a promising pharmacological approach to prevent endothelial dysfunction in cardiovascular diseases?. Pharmacol Ther.

[6] Morisseau C, Hammock BD (2013). Impact of soluble epoxide hydrolase and epoxyeicosanoids on human health. Annu Rev Pharmacol Toxicol.

[7] Bellien J, Iacob M, Remy-Jouet I, Lucas D, Monteil C, Gutierrez L, Vendeville C, Dreano Y, Mercier A, Thuillez C, Joannides R (2012). Epoxyeicosatrienoic acids contribute with altered NO and endothelin-1 pathways to conduit artery endothelial dysfunction in essential hypertension. Circulation.

[8] Zhang LN, Vincelette J, Chen D, Gless RD, Anandan SK, Rubanyi GM, Webb HK, MacIntyre DE, Wang YX (2011). Inhibition of soluble epoxide hydrolase attenuates endothelial dysfunction in animal models of diabetes, obesity and hypertension. Eur J Pharmacol.

[9] Gangadhariah MH, Dieckmann BW, Lantier L, Kang L, Wasserman DH, Chiusa M, Caskey CF, Dickerson J, Luo P, Gamboa JL, Capdevila JH, Imig JD, Yu C, Pozzi A, Luther JM (2017). Cytochrome P450 epoxygenase-derived epoxyeicosatrienoic acids contribute to insulin sensitivity in mice and in humans. Diabetologia.

[10] Duflot T, Pereira T, Roche C, Iacob M, Cardinael P, Hamza NE, Thuillez C, Compagnon P, Joannidès R, Lamoureux F, Bellien J (2017). A sensitive LC–MS/MS method for the quantification of regioisomers of epoxyeicosatrienoic and dihydroxyeicosatrienoic acids in human plasma during endothelial stimulation. Anal Bioanal Chem.

[11] Bellien J, Iacob M, Monteil C, Rémy-Jouet I, Duflot T, Thuillez C, Richard V, Joannidès R (2017). Physiological role of endothelin-1 in flow-mediated vasodilatation in humans and impact of cardiovascular risk factors. J Hypertens.

[12] Li D, Cui Y, Morisseau C, Gee SJ, Bever CS, Liu X, Wu J, Hammock BD, Ying Y (2017). Nanobody based immunoassay for human soluble epoxide hydrolase detection using polymeric horseradish peroxidase (PolyHRP) for signal enhancement: the rediscovery of PolyHRP?. Anal Chem.

[13] Borhan B, Mebrahtu T, Nazarian S, Kurth MJ, Hammock BD (1995). Improved radiolabeled substrates for soluble epoxide hydrolase. Anal Biochem.

[14] Enderle MD, Benda N, Schmuelling RM, Haering HU, Pfohl M (1998). Preserved endothelial function in IDDM patients, but not in NIDDM patients, compared with healthy subjects. Diabetes Care.

[15] Meyer MF, Lieps D, Schatz H, Pfohl M (2008). Impaired flow-mediated vasodilation in type 2 diabetes: lack of relation to microvascular dysfunction. Microvasc Res.

[16] Naka KK, Papathanassiou K, Bechlioulis A, Kazakos N, Pappas K, Tigas S, Makriyiannis D, Tsatsoulis A, Michalis LK (2012). Determinants of vascular function in patients with type 2 diabetes. Cardiovasc Diabetol.

[17] Modena MG, Bonetti L, Coppi F, Bursi F, Rossi R (2002). Prognostic role of reversible endothelial dysfunction in hypertensive postmenopausal women. J Am Coll Cardiol.

[18] Ghiadoni L, Versari D, Magagna A, Kardasz I, Plantinga Y, Giannarelli C, Taddei S, Salvetti A (2007). Ramipril dose-dependently increases nitric oxide availability in the radial artery of essential hypertension patients. J Hypertens.

[19] Cosentino F, Hishikawa K, Katusic ZS, Lüscher TF (1997). High glucose increases nitric oxide synthase expression and superoxide anion generation in human aortic endothelial cells. Circulation.

[20] Adela R, Nethi SK, Bagul PK, Barui AK, Mattapally S, Kuncha M, Patra CR, Reddy PN, Banerjee SK (2015). Hyperglycaemia enhances nitric oxide production in diabetes: a study from South Indian patients. PLoS ONE.

[21] McVeigh GE, Brennan GM, Johnston GD, McDermott BJ, McGrath LT, Henry WR, Andrews JW, Hayes JR (1992). Impaired endothelium-dependent and independent vasodilation in patients with type 2 (non-insulin-dependent) diabetes mellitus. Diabetologia.

[22] Heitzer T, Krohn K, Albers S, Meinertz T (2000). Tetrahydrobiopterin improves endothelium-dependent vasodilation by increasing nitric oxide activity in patients with type II diabetes mellitus. Diabetologia.

[23] Minuz P, Jiang H, Fava C, Turolo L, Tacconelli S, Ricci M, Patrignani P, Morganti A, Lechi A, McGiff JC (2008). Altered release of cytochrome p450 metabolites of arachidonic acid in renovascular disease. Hypertension.

[24] Issan Y, Hochhauser E, Guo A, Gotlinger KH, Kornowski R, Leshem-Lev D, Lev E, Porat E, Snir E, Thompson CI, Abraham NG, Laniado-Schwartzman M (2013). Elevated level of pro-inflammatory eicosanoids and EPC dysfunction in diabetic patients with cardiac ischemia. Prostaglandins Other Lipid Mediat.

[25] Makita K, Takahashi K, Karara A, Jacobson HR, Falck JR, Capdevila JH (1994). Experimental and/or genetically controlled alterations of the renal microsomal cytochrome P450 epoxygenase induce hypertension in rats fed a high salt diet. J Clin Invest.

[26] Wang MH, Smith A, Zhou Y, Chang HH, Lin S, Zhao X, Imig JD, Dorrance AM (2003). Downregulation of renal CYP-derived eicosanoid synthesis in rats with diet-induced hypertension. Hypertension.

[27] Furukawa M, Nishimura M, Ogino D, Chiba R, Ikai I, Ueda N, Naito S, Kuribayashi S, Moustafa MA, Uchida T, Sawada H, Kamataki T, Funae Y, Fukumoto M (2004). Cytochrome p450 gene expression levels in peripheral blood mononuclear cells in comparison with the liver. Cancer Sci.

[28] Huang H, Morisseau C, Wang J, Yang T, Falck JR, Hammock BD, Wang MH (2007). Increasing or stabilizing renal epoxyeicosatrienoic acid production attenuates abnormal renal function and hypertension in obese rats. Am J Physiol Renal Physiol.

[29] De Taeye BM, Morisseau C, Coyle J, Covington JW, Luria A, Yang J, Murphy SB, Friedman DB, Hammock BB, Vaughan DE (2010). Expression and regulation of soluble epoxide hydrolase in adipose tissue. Obesity (Silver Spring).

[30] Liu Y, Dang H, Li D, Pang W, Hammock BD, Zhu Y (2012). Inhibition of soluble epoxide hydrolase attenuates high-fat-diet-induced hepatic steatosis by reduced systemic inflammatory status in mice. PLoS ONE.

[31] Ai D, Fu Y, Guo D, Tanaka H, Wang N, Tang C, Hammock BD, Shyy JY, Zhu Y (2007). Angiotensin II up-regulates soluble epoxide hydrolase in vascular endothelium in vitro and in vivo. Proc Natl Acad Sci USA.

[32] Poreba M, Rostoff P, Siniarski A, Mostowik M, Golebiowska-Wiatrak R, Nessler J, Undas A, Gajos G (2018). Relationship between polyunsaturated fatty acid composition in serum phospholipids, systemic low-grade inflammation, and glycemic control in patients with type 2 diabetes and atherosclerotic cardiovascular disease. Cardiovasc Diabetol.

[33] Poreba M, Mostowik M, Siniarski A, Golebiowska-Wiatrak R, Malinowski KP, Haberka M, Konduracka E, Nessler J, Undas A, Gajos G (2017). Treatment with high-dose n-3 PUFAs has no effect on platelet function, coagulation, metabolic status or inflammation in patients with atherosclerosis and type 2 diabetes. Cardiovasc Diabetol.

[34] Ward NC, Rivera J, Hodgson J, Puddey IB, Beilin LJ, Falck JR, Croft KD (2004). Urinary 20-hydroxyeicosatetraenoic acid is associated with endothelial dysfunction in humans. Circulation.

[35] Steinberg HO, Brechtel G, Johnson A, Fineberg N, Baron AD (1994). Insulin-mediated skeletal muscle vasodilation is nitric oxide dependent. A novel action of insulin to increase nitric oxide release. J Clin Invest.

[36] Baron AD, Steinberg HO, Chaker H, Leaming R, Johnson A, Brechtel G (1995). Insulin-mediated skeletal muscle vasodilation contributes to both insulin sensitivity and responsiveness in lean humans. J Clin Invest.

[37] Hou HH, Hammock BD, Su KH, Morisseau C, Kou YR, Imaoka S, Oguro A, Shyue SK, Zhao JF, Lee TS (2012). N-terminal domain of soluble epoxide hydrolase negatively regulates the VEGF-mediated activation of endothelial nitric oxide synthase. Cardiovasc Res.

[38] Hou HH, Liao YJ, Hsiao SH, Shyue SK, Lee TS (2015). Role of phosphatase activity of soluble epoxide hydrolase in regulating simvastatin-activated endothelial nitric oxide synthase. Sci Rep.

[39] Williams SB, Goldfine AB, Timimi FK, Ting HH, Roddy MA, Simonson DC, Creager MA (1998). Acute hyperglycemia attenuates endothelium-dependent vasodilation in humans in vivo. Circulation.

[40] Kawano H, Motoyama T, Hirashima O, Hirai N, Miyao Y, Sakamoto T, Kugiyama K, Ogawa H, Yasue H (1999). Hyperglycemia rapidly suppresses flow-mediated endothelium-dependent vasodilation of brachial artery. J Am Coll Cardiol.

[41] Title LM, Cummings PM, Giddens K, Nassar BA (2000). Oral glucose loading acutely attenuates endothelium-dependent vasodilation in healthy adults without diabetes: an effect prevented by vitamins C and E. J Am Coll Cardiol.

[42] Beckman JA, Goldfine AB, Gordon MB, Creager MA (2001). Ascorbate restores endothelium-dependent vasodilation impaired by acute hyperglycemia in humans. Circulation.

[43] Arcaro G, Cretti A, Balzano S, Lechi A, Muggeo M, Bonora E, Bonadonna RC (2002). Insulin causes endothelial dysfunction in humans: sites and mechanisms. Circulation.

[44] Campia U, Sullivan G, Bryant MB, Waclawiw MA, Quon MJ, Panza JA (2004). Insulin impairs endothelium-dependent vasodilation independent of insulin sensitivity or lipid profile. Am J Physiol Heart Circ Physiol.

[45] Weintraub NL, Fang X, Kaduce TL, VanRollins M, Chatterjee P, Spector AA (1997). Potentiation of endothelium-dependent relaxation by epoxyeicosatrienoic acids. Circ Res.

[46] Hu J, Dziumbla S, Lin J, Bibli SI, Zukunft S, de Mos J, Awwad K, Frömel T, Jungmann A, Devraj K, Cheng Z, Wang L, Fauser S, Eberhart CG, Sodhi A, Hammock BD, Liebner S, Müller OJ, Glaubitz C, Hammes HP, Popp R, Fleming I (2017). Inhibition of soluble epoxide hydrolase prevents diabetic retinopathy. Nature.

[47] Luria A, Bettaieb A, Xi Y, Shieh GJ, Liu HC, Inoue H, Tsai HJ, Imig JD, Haj FG, Hammock BD (2011). Soluble epoxide hydrolase deficiency alters pancreatic islet size and improves glucose homeostasis in a model of insulin resistance. Proc Natl Acad Sci USA.

