# Supplementary material for: Altered bioavailability of epoxyeicosatrienoic acids is associated with conduit artery endothelial dysfunction in type 2 diabetic patients
Source: Cardiovasc Diabetol. 2019 Mar 18;18:35. doi: 10.1186/s12933-019-0843-z (PMC6423843; doi:10.1186/s12933-019-0843-z)
Supplement: Supplementary file 1 — Additional file 1. Supplementary Methods and Results. [file 12933_2019_843_MOESM1_ESM.doc]

Additional file 1

## Additional Methods

**Assessment of endothelial function**

Measurements were performed in the morning after a fat-free breakfast without tea or coffee for the comparative approach and under fasting conditions for the metabolic approach, while subjects were in a supine position, in a quiet air-conditioned room (22°C to 24°C). Subjects were asked to refrain from smoking from the previous evening. Radial internal diameter and blood flow velocity were continuously measured on the non dominant arm, using a high-precision echotracking device (NIUS 02, Asulab) coupled to a Doppler system (Doptek, Deltex). Radial artery flow was calculated from the measurement of blood velocity and diameter. Total blood viscosity was measured using a cone-plate viscometer (Ex100 CTB, Brookfield). From the individual values of radial artery diameter (d), flow (Q), and total blood viscosity (µ), the mean arterial wall shear stress, representing the tangential force due to the flowing blood acting on the endothelium, was calculated based on a Poiseuillean model *i.e.*, τ=[(4µQ)/(πr^3^), (r=d/2)].

Radial artery endothelium-dependent flow-mediated dilatation was assessed using hand skin heating. The hand skin temperature was modified by use of a water-filled thermo-controlled device (Polystat 1, Bioblock Scientific). Briefly, the hand was introduced in the thermo-controlled tank by use of a thin watertight glove fixed to the device. The device was then filled with water and the temperature was set to 34°C for 20 min. Then, hand skin heating was performed by increasing the water temperature from 34 to 37, 40, and 44°C with each level of temperature maintained for 7 min.

**Biological parameters**

##### Quantification of substrates and products of sEH

Plasma levels of EETs, DHETs, EpOMEs, DiHOMEs, 19,20-EpDPA and 19,20-DiHDPA were determined by high performance liquid chromatography coupled to tandem mass spectrometry after protein precipitation, lipids extraction and saponification.^1^ Briefly, 300 µL of plasma were spiked with 10 µL of internal standards (14,15-EETd_8_ and 11,12-DHETd_11_ at 200 ng/mL) and 1 mL of methanol was added for protein precipitation. After centrifugation, the supernatant was collected, acidified with 100 µL of formic acid 10%, and lipids were extracted by adding 1 mL of dichloromethane. The organic layer was collected and evaporated to dryness under a gentle stream of nitrogen. The resulting crude extract was saponified with sodium hydroxide at +90°C for 20 min to release bound fatty acids into their free form. After cooling on ice, the sample was acidified with formic acid and EETs and DHETs were extracted with 2mL of dichloromethane. The organic layer was collected and was evaporated to dryness, and reconstituted in 100 µL of methanol. Chromatographic separation was performed on a Kinetex C18 column (2.6-μm particle size, 50-mm length × 3-mm inner diameter). The auto-sampler temperature was set at 8 °C, the column oven at 30 °C, the injected volume was 20 μL, and the flow rate was 600 μL/min. The mobile phase was 0.2% formic acid in pure MeOH (solvent A) and 2 mM ammonium formate with 0.2% formic acid in water (solvent B). For EETs and DHETs, the elution was started with 95% B (0-0.5 min), 95-20% B (0.5-1 min), 20-10% B (1-4 min)10-5% B (4-5 min), 5-95% B (5-6 min), 95% B (6-7.5 min). For EpOMEs, DiHOMEs, 19,20-EpDPA and 19,20-DiHDPA, the flow rate was 500 μL/min and the following elution gradient was performed: 70% B (0-0.5 min), 70-35% B (0.5-1min), 35-15% B (1-11 min), 15-2% B (11-11.5 min), 2% B (11.5-13.5 min), 2-70% B (13.5-14min), 70% B (14-16min). Mass transitions (m/z) monitored in negative ion mode and mass spectrometer parameters are summarized in the Supplementary table S5.

### Quantification of plasma nitrite

Nitrite plasma level was determined using a tri-iodide/ozone-based chemiluminescence assay. Thawed plasma (250 µL) was injected into a glass purge vessel containing acidified tri-iodide solution, at room temperature, and actively purged with nitrogen in-line with a gas-phase chemiluminescence NO analyzer (NO Analyzer 280, Sievers). The tri-iodide solution rapidly reduces NO metabolites, mainly nitrite (>95% in plasma), to NO gas, which is measured by its gas-phase chemiluminescent reaction with ozone:

NO + O_3_ ⭢ NO_2_^*^ + O_2_

NO_2_^*^ ⭢ NO_2_+ hυ

The excited state of nitrogen dioxide emits light, which is then detected by a photomultiplier tube. Quantification of nitrite in a sample involves the conversion of the NO Analyzer signal into actual concentration using a calibration curve drawn from standard nitrite solutions prepared in phosphate buffer.

### Quantification of reactive oxygen species (ROS)

ROS level was evaluated in whole blood by electron paramagnetic resonance spectroscopy (EPR), using the spin probe 1-hydroxy-3-methoxycarbonyl-2,2,5,5-tetramethylpyrrolidine (CMH, Noxygen). Stock solution of CMH (50 mmol/L) was prepared in a Krebs-HEPES buffer (NaCl 0.1 mol/L, KCl 5 mmol/L, CaCl2 2.5 mmol/L, MgSO4 1.2 mmol/L, NaHCO3 25 mmol/L, KH2PO4 1 mmol/L, D(+)-Glucose 5.6 mmol/L, Na-HEPES 20 mmol/L, pH 7.4) containing defferoxamine 25 µmol/L and diethyldithiocarbamate 5 µmol/L. This stock solution of CMH was deoxygenated prior to the experiment in order to avoid CMH oxidation. Thus, 10 µL of CMH solution was previously placed into each prechilled 1 mL-syringe used for blood samples allowing it to be immediately mixed with the 600 µL blood collected. The syringe was immediately frozen in liquid nitrogen and kept at –80°C until analysis. The oxidation of CMH into the paramagnetic nitroxyde CM., driven mainly by superoxide reactions, was recorded on frozen sample using a MiniScope MS-200 X-band spectrometer (Magnettech). The EPR instrumental setting for field scan were as follows: Bo-field 3357 G, microwave power 1 mW, microwave frequency 9.74 GHz, modulation amplitude 5 G, sweep time 60 s, field sweep 100 G. The radical CM• concentration was calculed by comparing the signal intensity with that of a standard solution of CP• and expressed in µmol per liter of total blood.

##### Quantitation of sEH protein level

The sEH protein expression level was measured in PBMCs using an ultrasensitive polymeric horseradish peroxidase-based immunoassay. Briefly, the high-binding microplate (Nunc Cat.No. 442404) was coated with anti-human sEH rabbit serum (1:2000 dilution) in 0.05 M pH 9.6 carbonate-bicarbonate buffer (100 μL/well) overnight at 4 °C. After washing, the plate was blocked with 3% (w/v) skim milk (300 μL/well) in PBS for 1 h and washed before sample application. Serial concentrations of human sEH standards or samples with different dilutions in PBS containing 0.1 mg/mL bovine serum albumin (BSA) were then added to the wells (100 μL/well). To minimize the variation of incubation, human sEH standards and samples were first loaded into a 2-mL 96 deep well microplate (Costar 3960), and then transferred to the microplate for immunoassay within 1 min using multi-channel pipette. Immediately, biotinylated nanobodies (1 μg/mL, 100 μL/well) in PBS was added to each well. The immunoreaction was allowed to proceed for 1 h. After washing, SA-PolyHRP in PBS (25 ng/mL, 100 μL/well) was added and the reaction continued for another 30 min. After the final washing, 3,3′,5,5′-tetramethylbenzidine (TMB) substrate (100 μL/well) was added and the plate was incubated for 10-15 min. After stopping the color development with 2 M sulfuric acid (100 μL/well), the optical density was recorded at 450 nm within 10 min. All incubations unless otherwise specified were performed at room temperature with shaking (600 rpm); each washing step involved three washings with PBS containing 0.05% Tween-20 (PBST, 300 μL/well). Assays were performed in triplicate and the limit of detection is 0.046 ng/mL.

###### Determination of soluble epoxide hydrolase (sEH) activity

Peripheral blood mononuclear cells (PBMCs) were isolated by density gradient centrifugation with Ficoll-Paque (GE Healthcare Bio-Sciences AB). The cell suspensions were flash-frozen and kept at -80°C until analysis. After thawing on ice, the cells were broken with a 10 seconds ultrasonic pulse. Protein concentration was quantified using the Pierce BCA assay (Pierce, Rockford, IL), using Fraction V bovine serum albumin (BSA) as the calibrating standard. Separately, the homogenized mixture was diluted with chilled sodium phosphate buffer (20mM pH 7.4) containing 5 mM EDTA, 0. 1 mM DTT, 1 mM PMSF, 0.1 mg/mL BSA and 0.01% tween 20 to measure the residual sEH activity using [^3^H]-*trans*-diphenyl-propene oxide as substrate. Briefly, 1 µL of a 5 mM solution of t-DPPO in DMSO was added to 100 µL of diluted homogenate ([S]_final_ = 50 µM). The mixture was incubated at 37°C for 90 min, and the reaction quenched by addition of 60 µL of methanol and 200 µL of isooctane, which extracts the remaining epoxide from the aqueous phase. Extractions of the stopped reaction with 1-hexanol were performed in parallel to assess the possible presence of gluthation transferase activity which could also transform the substrate.^4^ The activity was followed by measuring the quantity of radioactive diol formed in the aqueous phase using a scintillation counter (TriCarb 2810 TR, Perkin Elmer, Shelton, CT). Assays were performed in triplicate and the limit of detection is 2.6 ng/mL.

## Additional Tables

**Table S1.** Primers used for quantitative RT-PCR.

|  | **Forward (5'-3')** | **Reverse (3'-5')** |
| --- | --- | --- |
| sEH | AAGATTTAGCCAGTGGCGTGTC | ATCACTGCTGGCAAAAGAACG |
| CYP2C9 | CTTGGAAAACACTGCAGTTGAC | TCCTGGACTTTAGCTGTGACC |
| CYP2C19 | TGGACATCAACAACCCTCGG | AGTCAGCTGCAGTGATTACCA |
| Beta2-microglobulin | CCACTGAAAAAGATGAGTATGCCT | CCAATCCAAATGCGGCATCTTCA |

sEH: soluble epoxide hydrolase.

**Table S2.** Radial artery diameter and mean wall shear stress assessed at 34°C before hand skin heating.

| Group | **Healthy (n=36)** | **Hypertension (n=9)** | **Type 2 diabetes**  **(n=10)** | **Type 2 diabetes + hypertension**  **(n=19)** |
| --- | --- | --- | --- | --- |
| Radial artery diameter, mm | 2.32±0.33 | 2.30±0.19 | 2.31±0.32 | 2.31±0.34 |
| Radial artery mean wall shear stress, dynes/cm² | 5.6±2.8 | 5.4±2.8 | 5.0±3.4 | 5.8±2.6 |

Values are mean±SD.

**Table S3.**

a. Stepwise models of the predictors of radial artery flow-mediated dilatation, mean wall shear stress variation during heating and GTN-induced dilatation.

| **Variable** | **Model** | **Predictors** | **AIC** | **Adjusted R Square** |
| --- | --- | --- | --- | --- |
| **Flow-mediated dilatation** | 1 | None | 218.44 | - |
|  | 2 | GTN | 188.47 | 0.374 |
|  | 3 | GTN + Group | 176.65 | 0.499 |
|  | 4 | GTN + Group + Mean wall shear stress variation | 175.73 | 0.513 |
|  | Final | GTN + Group + Mean wall shear stress variation + LDL cholesterol | 175.03 | 0.524 |
| **Mean wall shear stress variation** | 1 | None | 271.04 | - |
|  | 2 | Sex | 255.60 | 0.204 |
|  | 3 | Sex + Age | 253.28 | 0.239 |
|  | Final | Sex + Age + Smoke status | 250.36 | 0.289 |
| **GTN-induced dilatation** | 1 | None | 266.7 | - |
|  | 2 | Baseline diameter | 257.73 | 0.140 |
|  | 3 | Baseline diameter + Group | 251.57 | 0.249 |
|  | Final | Baseline diameter + Group + LDL cholesterol | 251.02 | 0.266 |

AIC: Akaike information criterion; GTN: glyceryl trinitrate-induced dilatation (per cent change in baseline diameter).

b. Predictors coefficients obtained from the final model

| **Variable** | **Predictor** | **Category** | **Adjusted coefficient**  **(±SE)** | **Adjusted P-value** |
| --- | --- | --- | --- | --- |
| **Flow-mediated dilatation** | Intercept | - | 3.61±2.71 | - |
|  | GTN | Per % increase | 0.33±0.07 | <0.001 |
|  | Group | Healthy *vs.* HT | -3.79±1.43 | 0.010 |
|  |  | Healthy *vs*.T2D | -3.26±1.38 | 0.022 |
|  |  | Healthy *vs.* HT + T2D | -3.52±1.19 | 0.004 |
|  | Mean wall shear stress variation | Per dynes/cm² increase | 0.12±0.07 | 0.103 |
|  | LDL cholesterol | Per g/L increase | 1.97±1.25 | 0.122 |
| **Mean wall shear stress variation** | Intercept | - | 24.34±4.86 | - |
|  | Sex | Man *vs.* Woman | 6.65±1.36 | <0.001 |
|  | Age | Per year increase | -0.21±0.08 | 0.017 |
|  | Smoke status | Never *vs.* past | 2.49±1.53 | 0.107 |
|  |  | Never *vs.* current | -5.85±3.30 | 0.081 |
| **GTN-induced dilatation** | Intercept | - | 54.02±7.17 | - |
|  | Baseline Diameter | Per *mm* increase | -9.37±2.67 | <0.001 |
|  | Group | Healthy *vs.* HT | 1.99±2.54 | 0.436 |
|  |  | Healthy *vs.*T2D | -6.62±2.34 | 0.006 |
|  |  | Healthy *vs.* HT + T2D | -2.02±2.11 | 0.343 |
|  | LDL cholesterol | Per g/L increase | 3.39±2.20 | 0.130 |

GTN: Glyceryl trinitrate-induced dilatation (percent change in baseline diameter); HT: hypertension; T2D: type 2 diabetes.

**Table S4.** Radial artery parameters and systemic hemodynamics assessed at 34°C before hand skin heating during saline infusion, hyperglycemic and hyperinsulinemic euglycemic clamps in 8 healthy subjects.

| **Group** | **Saline** | **Hyperglycemic clamp** | **Hyperinsulinemic clamp** |
| --- | --- | --- | --- |
| Radial artery diameter, mm | 2.35±0.22 | 2.46±0.25 | 2.48±0.30* |
| Radial artery mean wall shear stress, dynes/cm² | 3.9±1.5 | 5.2±0.9 | 4.6±1.8 |
| Mean arterial pressure, mmHg | 127±11 | 133±6 | 133±14 |
| Heart rate, bpm | 56±7 | 59±7 | 60±7 |

Values are mean±SD.**P*<0.05 *vs.* saline.

**Table S5.** LC-MS/MS parameters for EETs, DHETs and internal standards.

| **Analyte** | **Mass transition** | |  | **MS parameters** | | | **Internal standard (IS)** |
| --- | --- | --- | --- | --- | --- | --- | --- |
|  | **m/z (MS1)** | **m/z (MS3)** |  | **DP (V)** | **CE (eV)** | **CXP (V)** |  |
| 14,15-DHET | 337.1 | 207.1 |  | -90 | -24 | -9 | 11,12-DHET-d_11_ |
| 11,12-DHET | 337.1 | 167.0 |  | -90 | -26 | -9 | 11,12-DHET-d_11_ |
| 8,9-DHET | 337.1 | 185.0 |  | -80 | -22 | -15 | 11,12-DHET-d_11_ |
| 14,15-EET | 319.1 | 219.1 |  | -90 | -16 | -9 | 14,15-EET-d_8_ |
| 11,12-EET | 319.1 | 207.9 |  | -70 | -16 | -9 | 14,15-EET-d_8_ |
| 8,9-EET | 319.1 | 126.9 |  | -65 | -24 | -9 | 14,15-EET-d_8_ |
| 12,13-DiHOME | 313.1 | 183.2 |  | -100 | -30 | -5 | 11,12-DHET-d_11_ |
| 9,10-DiHOME | 313.1 | 201.0 |  | -110 | -30 | -7 | 11,12-DHET-d_11_ |
| 12,13-EpOME | 295.4 | 195.0 |  | -90 | -22 | -15 | 14,15-EET-d_8_ |
| 9,10-EpOME | 295.4 | 171.0 |  | -105 | -22 | -13 | 14,15-EET-d_8_ |
| 19,20-DiHDPA | 361.0 | 273.1 |  | -100 | -28 | -11 | 11,12-DHET-d_11_ |
| 19,20-EpDPA | 343.1 | 299.2 |  | -85 | -14 | -9 | 14,15-EET-d_8_ |
| 11,12-DHET-d_11_ | 348.1 | 166.9 |  | -135 | -28 | -5 |  |
| 14,15-EET-d_8_ | 327.1 | 226.0 |  | -90 | -18 | -18 |  |

## Additional Figures


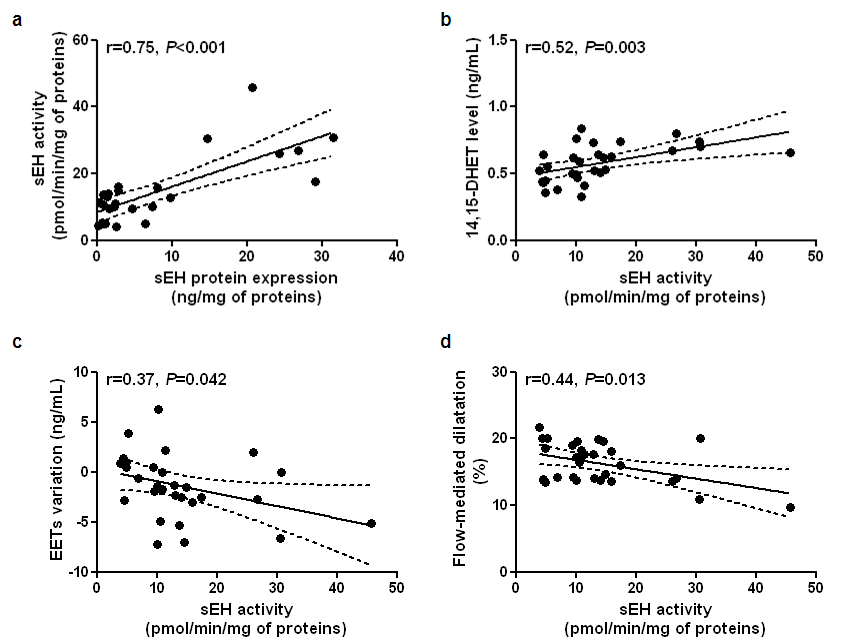


**Figure S1.** Linear relationship between soluble epoxide hydrolase (sEH) protein expression with sEH activity (a) and between sEH activity with the plasma level of 14,15-dihydroxyeicosatrienoic acid at the end of heating (14,15-DHET; b), epoxyeicosatrienoic acids (EETs) variation (c) and radial artery flow-mediated dilatation adjusted to shear stress variation (d). The dashed lines represent the 95% confidence interval for the regression.

**Figure S2.** Plasma levels of the he 19,20 epoxide of docosahexaenoic acid (19,20-EpDPA)and 9,10 and 12,13 epoxides of linoleic acid (9,10-EpOME and 12,13-EpOME) (a) and their respective diols produced by sEH, 19,20-DiHDPA, 9,10-DiHOME and 12,13-DiHOME (b) before (34°C) and at the end of hand skin heating (44°C), and corresponding ratios (c) determined in healthy, hypertensive (HT), type 2 diabetic (T2D) and hypertensive type 2 diabetic (HT+T2D) subjects. Mean values ±SEM are shown.


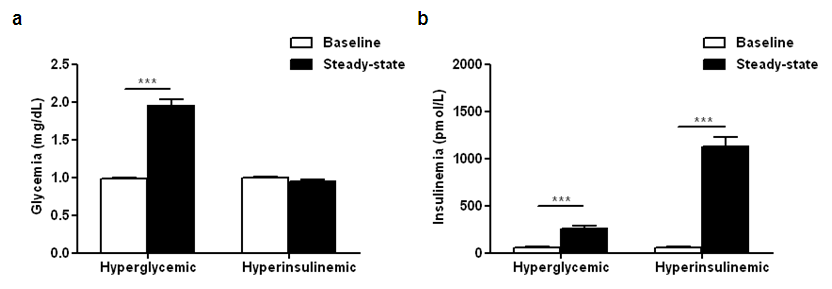


**Figure S3.** Plasma glucose (a) and insulin (b) concentrations before and at steady-state before hand skin heating during the hyperglycemic and hyperinsulinemic euglycemic clamps in 8 healthy subjects. Mean values ±SEM are shown. ****P*<0.001.


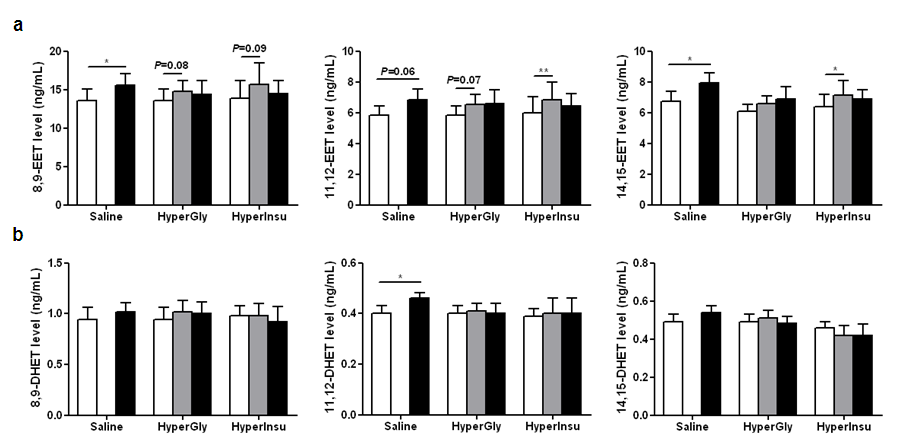


**Figure S4.** Plasma levels of epoxyeicosatrienoic acid (EETs) (a) and dihydroxyeicosatrienoic acids (DHETs) (b) regioisomers at baseline at 34°C, at steady-state and at the end of hand skin heating at 44°C during saline infusion and during the hyperglycemic (HyperGly) and hyperinsulinemic euglycemic (HyperInsu) clamps in 8 healthy subjects. Mean values ±SEM are shown. **P*<0.05, ***P*<0.01.


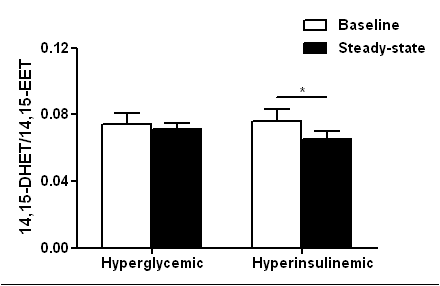


**Figure S5.** Ratio of plasma 14,15-dihydroxyeicosatrienoic acid-to-14,15-epoxyeicosatrienoic acid (14,15-DHET/14,15-EET) before and at steady-state before hand skin heating during the hyperglycemic and hyperinsulinemic euglycemic clamps in 8 healthy subjects. Mean values ±SEM are shown. **P*<0.05.

Figure S6. Schematic representation of the mechanisms involved in conduit artery endothelial dysfunction in essential hypertension and type 2 diabetes. There was a decrease in endothelium-dependent relaxation of vascular smooth muscle cells in treated essential hypertensive patients and type 2 diabetic patients. In essential hypertension, this decreased is not related to a modification in the production of NO by endothelial NO-synthase in response to shear stress, as shown by the similar increase in the NO metabolite nitrite (NO_2_^-^) levels compared to healthy subjects, but to an altered synthesis of epoxyeicosatrienoic acids (EETs) by endothelial cytochrome P450. The reduced flow-mediated dilatation in type 2 diabetic patients is due to a decreased NO bioavailability, which appears related to its breakdown by superoxide anions (O_2_^•-^), and decreased EETs levels due to altered synthesis and increased degradation to dihydroxyeicosatrienoic acids (DHETs) by soluble epoxide hydrolase (sEH), whose expression is upregulated.
